# Switching from blonanserin tablets to blonanserin transdermal patches improves tardive dyskinesia: A case report

**DOI:** 10.1002/npr2.12200

**Published:** 2021-08-06

**Authors:** Shintaro Sakai, Yoshiro Morimoto, Yusuke Matsuzaka, Takeshi Nakano, Shinji Kanegae, Akira Imamura, Hiroki Ozawa

**Affiliations:** ^1^ Department of Neuropsychiatry Unit of Translation Medicine Nagasaki University Graduate School of Biomedical Sciences Nagasaki Japan; ^2^ Child and Adolescent Psychiatry Community Partnership Unit Nagasaki University Hospital Nagasaki Japan

**Keywords:** blonanserin tablets, blonanserin transdermal patches, orofacial dyskinesia

## Abstract

Tardive dyskinesia (TD) is a common side effect of antipsychotics, and it remains a persistent and challenging problem. The blonanserin transdermal patch, developed in Japan and launched in September 2019, is the first antipsychotic transdermal treatment. Here, we describe a patient with schizophrenia who exhibited markedly improved orofacial dyskinesia after switching from blonanserin tablets to blonanserin transdermal patches. We speculate that the patch formulation might have led to more stable plasma blonanserin levels, thus reducing the side effects. Specifically, the patch formulation might have contributed to stable plasma levels via the continuous and direct absorption of blonanserin through the skin.

## Abstract 2

The present case report shows that switching from blonanserin tablets to blonanserin patches may reduce EPSs while maintaining a good therapeutic effect.

## Graphical abstract

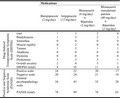

## 1 INTRODUCTION

Tardive dyskinesia (TD) is a common side effect of antipsychotics. It often causes physical and emotional distress in individuals and affects their quality of life.^1^ Despite the advent of atypical or second‐generation antipsychotics, which have a lower risk of complications, TD remains a persistent and challenging problem.^2^ Blonanserin is a second‐generation antipsychotic commonly used in Asian countries (eg, Japan and Korea) with a unique pharmacological profile.^3^ Compared with other second‐generation antipsychotics, it is characterized by higher dopamine D2 receptor occupancy and lower serotonin 2A receptor blockade,^4^ and improves not only positive symptoms of schizophrenia but also cognitive symptoms and some social functions.^5^, ^6^, ^7^, ^8^ The efficacy of blonanserin for schizophrenia is comparable to that of other antipsychotics, and it is well tolerated, with a lower risk of weight gain and hyperprolactinemia compared with other second‐generation antipsychotics.^7^, ^8^ The blonanserin transdermal patch (blonanserin tape), developed in Japan and launched in September 2019, is the first form of antipsychotic transdermal therapy in the world. It was developed as an alternative to blonanserin tablets to improve adherence and stabilize blood levels of the drug.^9^ Here, we describe a patient with schizophrenia who exhibited a marked improvement in orofacial dyskinesia after switching from blonanserin tablets to blonanserin transdermal patches. To the best of our knowledge, this is the first such case report.

## 2 CASE PRESENTATION

The patient was a 61‐year‐old woman with schizophrenia who had orofacial dyskinesia.

She had no previous physical problem and family history of psychiatric disorders. The patient was healthy during her school years. She graduated from nursing school and began to work at the age of 22. At the age of 43, several psychiatric symptoms, including insomnia, anxiety, and mood swings, began to appear. Aged 45, the patient started to experience auditory hallucinations, thought insertions, and delusions. She was diagnosed with schizophrenia and was prescribed olanzapine (1.25‐5 mg/d), which was effective against her symptoms. She continued to work as a nurse until she was 60 years old and then retired. However, at the age of 61, she refused medication and her psychotic symptoms worsened. She jumped off a bridge and fell into a river while experiencing an auditory hallucination, and was transported to hospital because of a whole‐body fracture. When her physical condition had improved, she was transferred to the psychiatric hospital, and inpatient psychiatric treatment was initiated. She first resumed treatment with olanzapine (5 mg/d), but this was discontinued because of hyperglycemia. Next, she was treated with paliperidone (6 mg/d; 2 weeks), but orofacial dyskinesia, which is classified as an extrapyramidal symptom (EPS), appeared. To alleviate the orofacial dyskinesia, her medication was switched from paliperidone to blonanserin (6‐8 mg/d; 8 months). With the administration of blonanserin, psychiatric symptoms were stabilized, and dyskinesia was reduced for several months. However, her orofacial dyskinesia gradually worsened and became a serious disturbance in her daily life (eg, liquid spills when she ate and drank, wearing a mask at all times because of her orofacial appearance). Blonanserin was discontinued because of her severe EPSs, and she was then treated with brexpiprazole (2 mg/d; 2 weeks). After switching to brexpiprazole, her hallucinations and delusions worsened (Positive and Negative Syndrome Scale [PANSS] = 78) and her EPSs continued (Drug‐Induced Extrapyramidal Symptoms Scale [DIEPASS] = 8, dyskinesia = 3). The patient was then switched from brexpiprazole to aripiprazole (12 mg/d; 2 weeks); however, the medications were discontinued because of her severe EPSs and psychiatric symptoms (PANSS = 89, DIEPASS = 18, dyskinesia = 4). Although we tried several antipsychotics (eg, aripiprazole and brexpiprazole), none of them were well tolerated and they did not provide an adequate therapeutic effect. A recent systematic review reported that switching to clozapine could reduce the risk of tardive dyskinesia and/or treat existing tardive dyskinesia.^10^ However, when we provided information about clozapine to the patient and her family, they decided against clozapine treatment due to concerns about side effects. For this reason, and because the previous treatment history showed that blonanserin had a substantial therapeutic effect, we prescribed a combination of blonanserin and anticholinergic drugs. Thus, the patient was again treated with blonanserin (8 mg/d; 7 weeks) as well as biperiden (2 mg/d; 7 weeks). After switching back to blonanserin, her severe EPSs—including orofacial dyskinesia—improved to some extent, but persisted (PANSS = 74, DIEPASS = 9, dyskinesia = 3). Finally, based on the striatal dopamine D2 receptor occupancy of blonanserin tablets and blonanserin transdermal patches in a clinical trial (Phase II PET study), blonanserin tablets (8 mg/d) were switched to blonanserin transdermal patches (40 mg/d).^11^ After this switch, the patient's orofacial dyskinesia markedly improved and she was discharged from the hospital (PANSS = 63, DIEPASS = 4, dyskinesia = 1). Detailed information about her medication history and EPSs is summarized in Table 1.

**TABLE 1:** Summary of the patient's medications and psychopathological/extrapyramidal symptoms

	Medications			
	Brexpiprazole (2 mg/d)	Aripiprazole (12 mg/d)	Blonanserin (8 mg/d) + Biperiden (2 mg/d)	Blonanserin transdermalpatches (40 mg/d) + Biperiden (2 mg/d)
Drug‐induced extrapyramidal symptoms scale (DIEPSS)				
Gait	1	3	2	0
Bradykinesia	1	3	2	1
Sialorrhea	0	0	0	0
Muscle rigidity	0	2	0	0
Tremor	1	3	1	1
Akathisia	0	0	0	0
Dystonia	0	0	0	0
Dyskinesia	3	3	3	1
Overall severity	2	4	3	1
DIEPSS (total)	8	18	11	4
Positive and negative syndrome scale (PANSS)				
Positive scale	24	22	20	17
Negative scale	20	24	21	18
General psychopathology scale	34	43	35	28
PANSS (total)	78	89	76	63

## 3 DISCUSSION

Antipsychotics, including blonanserin, can reduce symptoms of schizophrenia by acting on dopamine D2 receptors in the brain.^12^, ^13^ However, blonanserin is associated with a relatively high incidence of EPSs, and EPS incidence during treatment is mainly determined by the plasma concentration of antipsychotics.^14^ Iwata et al^15^ recently reported that the overall EPS incidence with blonanserin transdermal patches is lower than that of blonanserin tablets. Moreover, these authors suggested that the lower EPS incidence may be caused by more stable plasma blonanserin levels with the transdermal patch compared with oral formulations.^15^ The increased plasma level stability with the patch formulation may be related to the direct absorption of blonanserin through the skin. As the compound bypasses the gastrointestinal tract, no effects of food on bioavailability (as is the case with oral blonanserin) are observed.^16^ Furthermore, the blonanserin patch provides continuous release of blonanserin, which may also contribute to stable plasma blonanserin levels.^9^ It is therefore possible that, in the present patient, the stable blood levels made possible by the blonanserin patch resulted in both a favorable therapeutic effect and a reduction in side effects.

In summary, the present case report shows that switching from blonanserin tablets to blonanserin patches may reduce EPSs while maintaining a good therapeutic effect. By collecting data from more cases and studying the pharmacological properties of blonanserin patches, more appropriate treatments may be identified for schizophrenia patients. Although this treatment is currently only available in Japan, it is hoped that blonanserin patches will soon be available in additional countries.

## 4 INFORMED CONSENT

Informed consent was obtained from the participant.

## CONFLICT OF INTEREST

The authors declare no conflicts of interest associated with this article.

## AUTHOR CONTRIBUTIONS

SS treated the patient and drafted the manuscript. Yo.M. critically reviewed the draft and revised it. All authors made substantial contributions, drafted the manuscript, and approved the final manuscript.

## Data Availability

Data sharing is not applicable to this article as no datasets were generated or analyzed during the current study.
